# De novo construction of a “Gene-space” for diploid plant genome rich in repetitive sequences by an iterative Process of Extraction and Assembly of NGS reads (iPEA protocol) with limited computing resources

**DOI:** 10.1186/s13104-016-1903-z

**Published:** 2016-02-11

**Authors:** Christelle Aluome, Grégoire Aubert, Susete Alves Carvalho, Marie-Christine Le Paslier, Judith Burstin, Dominique Brunel

**Affiliations:** INRA Institut National de la Recherche Agronomique, US1279 Etude du Polymorphisme des génomes Végétaux, CEA-IG/CNG Centre National de Génotypage, 2 rue Gaston Crémieux, 91057 Evry, France; INRA Institut National de la Recherche Agronomique, UMR1347 Agroécologie, 17 rue Sully, 21065 Dijon Cedex, France

**Keywords:** Gene-space, Unigene, Next-generation sequencing NGS, Assembly, Iterative process, Limited computing resources

## Abstract

**Background:**

The continuing increase in size and quality of the “short reads” raw data is a significant help for the quality of the assembly obtained through various bioinformatics tools. However, building a reference genome sequence for most plant species remains a significant challenge due to the large number of repeated sequences which are problematic for a whole-genome quality de novo assembly. Furthermore, for most SNP identification approaches in plant genetics and breeding, only the “Gene-space” regions including the promoter, exon and intron sequences are considered.

**Results:**

We developed the iPea protocol to produce a de novo Gene-space assembly by reconstructing, in an iterative way, the non-coding sequence flanking the Unigene cDNA sequence through addition of next-generation DNA-seq data. The approach was elaborated with the large diploid genome of pea (*Pisumsativum* L.), rich in repetitive sequences. The final Gene-space assembly included 35,400 contigs (97 Mb), covering 88 % of the 40,227 contigs (53.1 Mb) of the PsCam_low-copy Unigen set. Its accuracy was validated by the results of the built GenoPea 13.2 K SNP Array.

**Conclusion:**

The iPEA protocol allows the reconstruction of a Gene-space based from RNA-Seq and DNA-seq data with limited computing resources.

**Electronic supplementary material:**

The online version of this article (doi:10.1186/s13104-016-1903-z) contains supplementary material, which is available to authorized users.

## Background

Next-generation sequencing (NGS) technologies and their low cost provide an easy access to the sequences of many genotypes and thus to the single nucleotide polymorphisms (SNPs). This ability has changed many applications of plant and animal genetics: analysis of genetic resources, QTL mapping, association genetics, marker-assisted breeding. The construction of genotyping array for the joint analysis of thousands or even hundreds of thousands SNP is a major challenge for the improvement of plant and animal species (association genetics, genomics selection, etc.).

However to identify the SNPs, the most commonly used bioinformatics methods require a mapping step performing the alignment of raw data (reads) to a reference sequence.

The continuing increase in size and quality of the “short reads” sequences is a significant help for the quality of the assembly provided by various bioinformatics tools (Velvet [1], ABySS [2], Bowtie [3], SOAPdenovo [4]), but simultaneously it causes increased costs in terms of computer processing [5]. Even though, for most plant species, building a reference genome sequence remains a significant challenge due to the large number of repeated sequences that are problematic for a quality de novo assembly. The constitution of international consortia is still necessary for mobilizing the technical and IT resources required.

In practice, for most of the applications mentioned above, only the sequence portion of the “Gene-space” (with gene implied in a broad sense as: promoter, exon and intron) is actually considered when identifying SNPs. The positioning of SNPs on repetitive sequences is de facto very difficult if not impossible because these sequences have multiple locations in the genome. The use as a reference sequence of a “Unigene”, built from a RNA-seq sequence data, is an effective and economical alternative. However, for SNP identification, the Unigene set is still limited by the fact that much of the variability between genotypes of crop species are found in the non-coding portion of the gene (intron sequences, 3′ and 5′ UTR), less subject to selection pressure.

We present here a bioinformatics approach which allows, for a diploid species without a complete genome reference sequence, the de novo assembly of a Gene-space combining DNA-seq data from high throughput sequencing and a Unigene sequence set built from RNA-seq data.

The approach was elaborated and tested for building a genotyping chip for pea (*Pisum sativum* L.). The pea genome is large with a genome estimated ca. 4.45 Gb, diploid (2n = 14) with full of repetitive elements, with 75–95 % of it being repeated sequences [6]. The efficiency of the de novo assembly obtained by this protocol was validated by different methods, and particularly by the results of the high throughput genotyping array built from it.

This de novo assembly protocol allows laboratories with limited IT resources to obtain a reference sequence consisting of a large part of the Gene-space using RNA-seq and DNA-seq data now easy accessible in any plant species.

## Methods

### General principle of the method

**Fig. 1:**
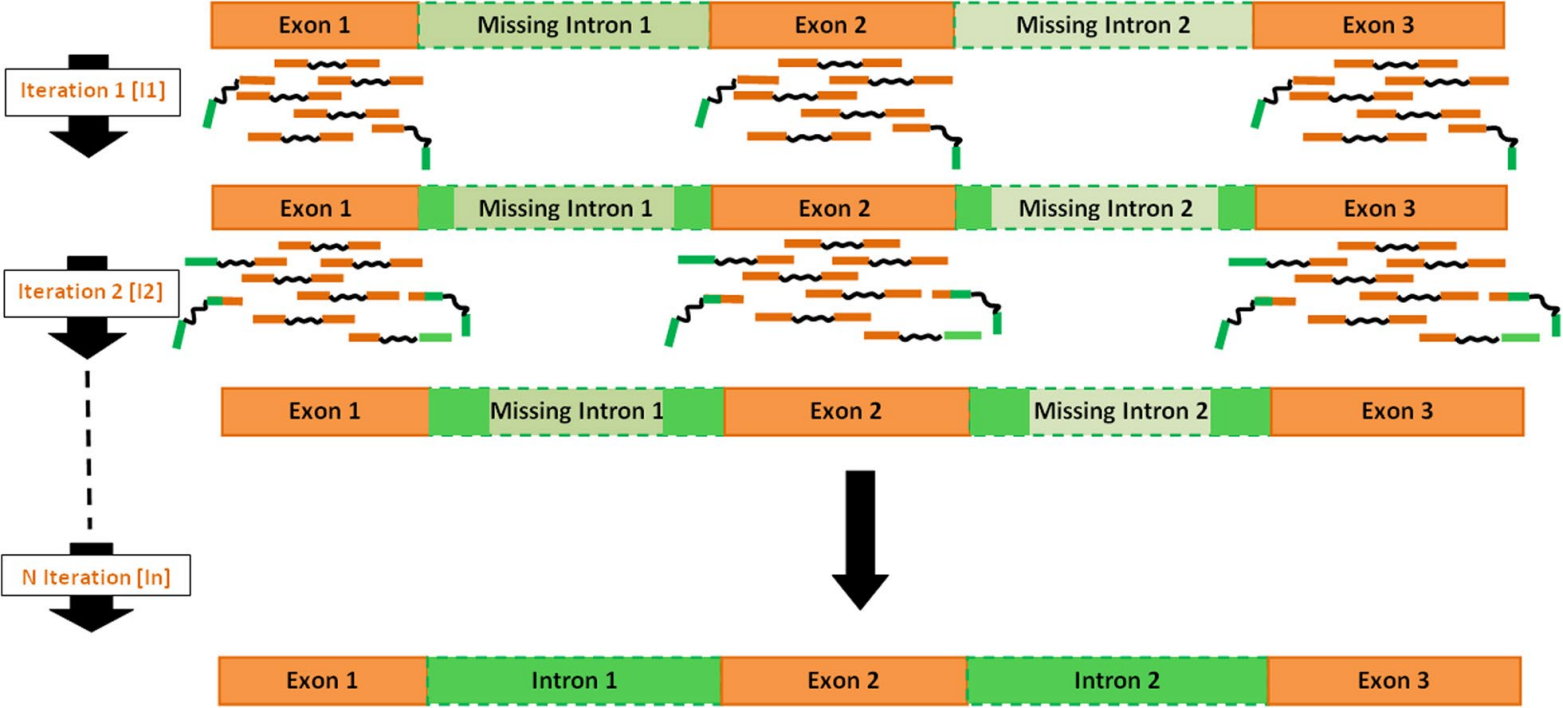
The evolution of the reconstruction of a genic sequence, represented by a succession of exons (*orange*) and intron (*green*). The paired-end reads are colored in *orange* or in *green* if they correspond to an intronic sequence (*green*), an exonic sequence (*orange*) or a junction intron/exon sequence (*green* and *orange*). The figure shows that during the iterations, the introns are added to the exons present in the first reference sequence (Unigene), providing additional information for the next iteration

**Fig. 2:**
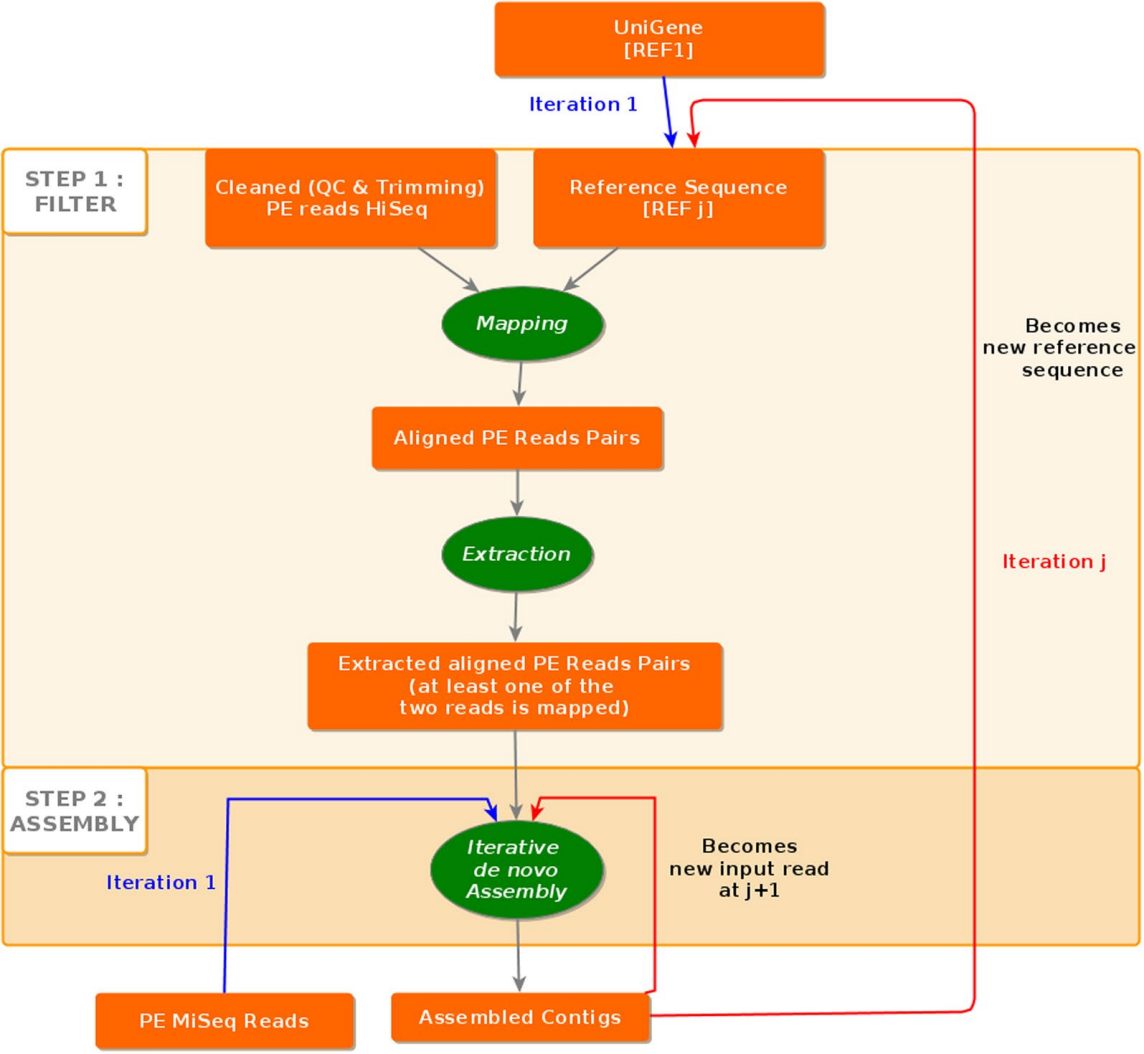
The method diagram. For the first iteration, HiSeq 2000 short reads were submitted as “input short read” while longer reads from MiSeq are submitted as “input long reads”. For further iteration Ij, the contigs produced by Ij-1 are used as a new “input long read”, in order to maintain the assembly already produced

**Fig. 3:**
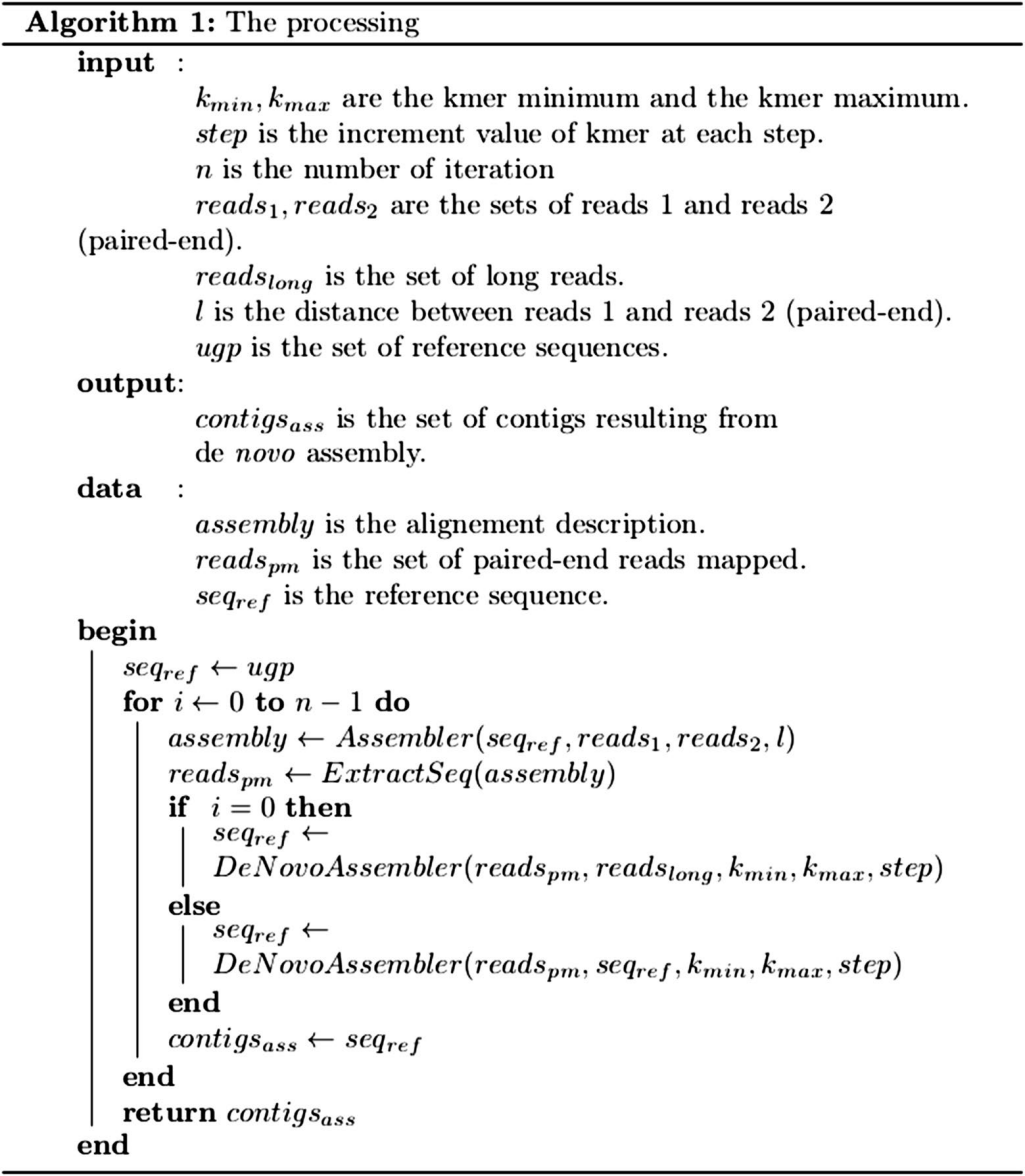
The algorithm of the processing

We used an iterative protocol (Figs. 1, 2, 3) where each iteration (I1 to In) included two steps. Step.1 implemented a “filter” that only retained the DNA-seq paired-end reads showing homology with a reference sequence file provided at each iteration. Step.2 assembled the paired-end reads filtered at Step.1 into contigs.

The “filtration Step.1” was performed by mapping paired-end reads against the reference sequence file provided (either Unigene contigs for the 1st iteration or the extended sequence from the previous iteration). The reads R1 and R2 are physically linked as the data were produced in “paired-end reads”. All pairs of reads of which at least one of the reads was mapped onto the reference were extracted for the Step.2. At the first iteration, the reference sequence is the Unigene, consisting of exon sequences. If only one read of one pair is mapped, it means that the second unmapped read is either in an intron (therefore absent from the reference sequence), or in an overlapping intron/junction. These unmapped reads (in green in Fig. 1) provide the missing intronic sequences at each later iterations.

The mapping and extraction tools used (CLC_ref_assemble_long and sub_assembly, respectively) are part of the CLC Assembly Cell Package (version 3.22). The mapping parameter for the distance between R1 and R2 corresponds to the insert lengths of the sequenced DNA libraries. The value of the coefficient identity is chosen very high because the reads obtained by sequencing are mapped to the Unigene built from the same genotype Caméor.

The “de novo assembly Step.2” reconstructed contigs from the filtered read pairs. This step was implemented through the HKU-IDBA tool (version 1.09). HKU-IDBA [7] was selected after comparison with Velvet [1] and ABySS [2]. HKU-IDBA requires less RAM or CPU resources and provides better results for our application. HKU-IDBA, based on De Bruijn graph, itself uses an iterative process. HKU-IDBA varies its value of k-mer between a minimum kmin and a maximum kmax for each iteration by incrementing a step value. It successively provides de novo assemblies for these k-mers, which allows to obtain a better result by correcting and validating its contigs. Using an iterative k-mer also overcomes the problem of choosing the k-mer value which is very important in this type of de novo assembly protocol [8]. The use of multiple k-mers should avoid the problem with even kmers that could become reverse complements of their own sequences [9]. HKU-IDBA inputs reads files with size less than or equal to 128 nt as “input short read” and files containing reads greater than 128 nt size as “input long read”. At the end of each iteration, a fasta file containing all contigs longer than a minimal threshold size defined by the user was produced. This output file is then used at the next iteration as a new reference sequence for the mapping (Step.1) and also as a new “input long read” for the HKU-IDBA tool (Step.2) in order to keep the assembly built at the previous iteration. The algorithm of the processing is described in Fig. 3.

### Application to the construction of the Pea Gene-space

Two TruSeq Illumina libraries of insert sizes 390 and 620 nt were produced from a unique total DNA of the inbred cultivar Caméor (provided by the Dijon team) and subjected to HiSeq 2000 (paired-end, read length = 101 nt) and MiSeq (paired-end, read length = 250 nt) sequencing, following the provider’s instructions. 562,493,396 and 28,527,820 raw reads were produced from HiSeq and Miseq sequencing, respectively. A data quality control on the HiSeq files, carried out with the software fastaQC [10], showed that for the majority of reads, the Phred score was between 30 and 40. A trimming was performed on the HiSeq files with the constraints defined as followed: a base is maintained if its Phred value is greater or equal to 30. The resulting read has a size greater or equal to 30 bases and without ambiguous base. 530,868,977 (94 %) paired-end reads were conserved (Additional file 1).

At the first iteration I1, the pea PsCam_LowCopy UniGene set [11], built from RNA-seq data from Caméor, containing 40,227 contigs in a fasta format, was used as the initial sequence. These contigs have sizes that vary between 203 and 16,601 nt, with an average size of 1277 nt and a N50 of 1725 nt for a total of 53.1 Mb. Mapping parameters in Step.1 were defined as follows: 95 % similarity between a read sequence and the reference sequence and a distance between read1 and read2 of 100–400 nt for short sizing libraries, or 250–600 nt for longer sizing libraries. In Step 2, the HKU-IDBA algorithm was used with the following parameters for all iterations: kmin value (–mink) of 20 nt, kmax (–maxk) of 100 nt, with an increment (–step) of 5nt, minimum contig value (–min_contig) of 200 nt, similarity value (–similar) of 1 nt.

For the first iteration, HiSeq 2000 short reads (smaller than 128 nt) were submitted as “input short read” while long reads of size greater than or equal to 128 nt from MiSeq are submitted as “input long reads”. For further iteration Ij, the contigs produced by Ij-1 are used as a new “input long read”, in order to maintain the assembly already produced (Figs. 2, 3).

A «standalone BLAST» [12] was performed to assess the quality of contigs from the assembly with the tools of NCBI-BLAST-2.2.29+, and more particularly MAKEBLASTDB and BLASTN [13]. The e-value chosen to 1–10^−60^.

All calculations were performed on a Dell PowerEdge R5610 2 × QUAD CORE XEON 2.66 GHz 96 GB RAM.

## Results

### The implementation of iPEA

The first filtration step was used to retrieve though mapping, all reads corresponding to or close to the coding sequences. The statistics of mapping for the first iteration I1 (Additional file 2) show that regardless of the library size, the percentage of reads mapped is still of the order of 1 %. Such a percentage is consistent with the estimate of coding sequences in the pea genome. The proportion of multi-hits reads (mapping at multiple locations of the reference sequence) is still on the order of 10 % which can indicate the presence of gene families in the Unigene.

The percentage of paired-end mapped reads was twice as large for the small insert library (26 % for the library with a 390 nt insert), than for the large insert library (13 % for the library with a 620 nt insert). Because the estimation of the mean sizes of the exons in *Lotus* and *Medicago* species are 127–140 nt, respectively [14], one reason could be that two reads of one pair come more often from the same exon in the libraries of short inserts than in the larger one. In the case of a library with a longer insert, more broken pairs are found, indicating that only one read is homologous to an exon, the second corresponding to a part of an intron sequence, not present in the Unigene.

At the first iteration, both the trimmed and mapped HiSeq 2000 reads (16,335,096 short read input, paired-ends, trimmed, 30–101 nt) and the untrimmed MiSeq reads (28,527,820 long read input, paired-ends, 250 nt), were assembled with the HKU-IDBA software. The MiSeq reads were not trimmed as the average sequencing depth was about 1.6×, but their main interest was their higher length that help to anchor the contigs together.

**Fig. 4:**
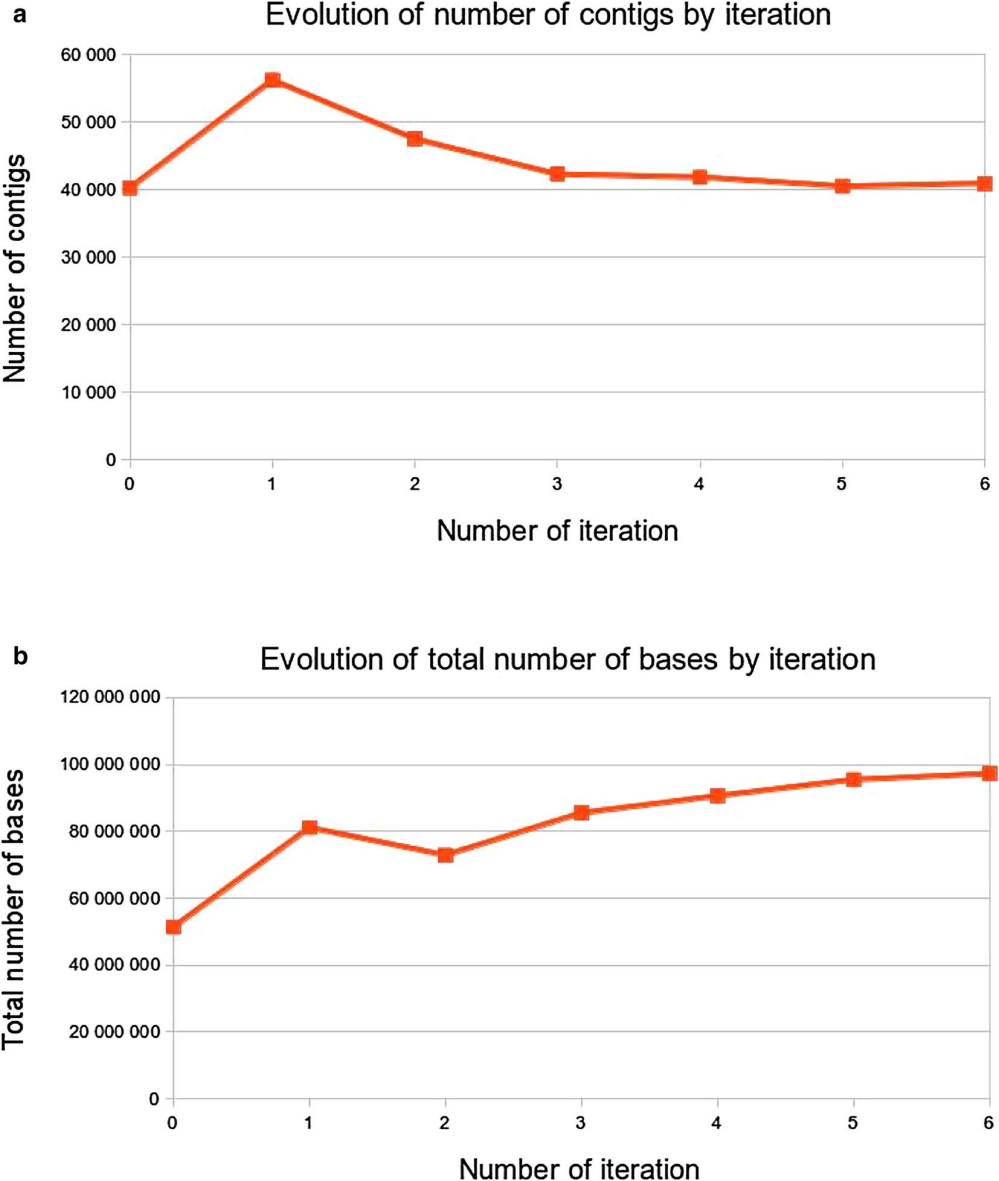
During the successive iterations, **a** the number of de novo genomics contigs decreases, **b** the mean of the de novo contig length increases, until reaching a plateau at the 6th iteration

The results of the different iterations 1–6 are shown in Fig. 4a, b and Additional file 3. During the successive iterations, the number of de novo contigs decreases from 56,219–40,901 with lengths between 299 and 18,907 nt, the mean of the de novo contig length increases from 1443 to 2378 nt and the entire assembly size increases from 53.1 (the Unigene size) to 97 Mb. The results indicate that each iteration provides additional reads obtained by the filtration step which can help to join the contigs. But after the six iterations, the number of contigs and the cumulative number of bases reached a plateau.

### Assesment of the iPEA assembly

For the validation of the de novo assembly, one of the most common metric is the N50 value, i.e. the contig size for which all larger contigs cover 50 % of the total length of the assembly [15, 16]. In our protocol, the N50 increases from 1931 (iteration I1) to 3416 (iteration I6) (Additional file 2) which is consistent with medium gene size in plants.

Because this metric is not sufficient to validate the consistency of contigs obtained, two other information allowed us to estimate the quality of the assembly: (1) the rate of reconstruction of genes compared to the Unigene and (2) the results of the GenoPea 13.2 K Illumina genotyping BeadChip constructed from the assembly obtained in the sixth iteration.

**Fig. 5:**
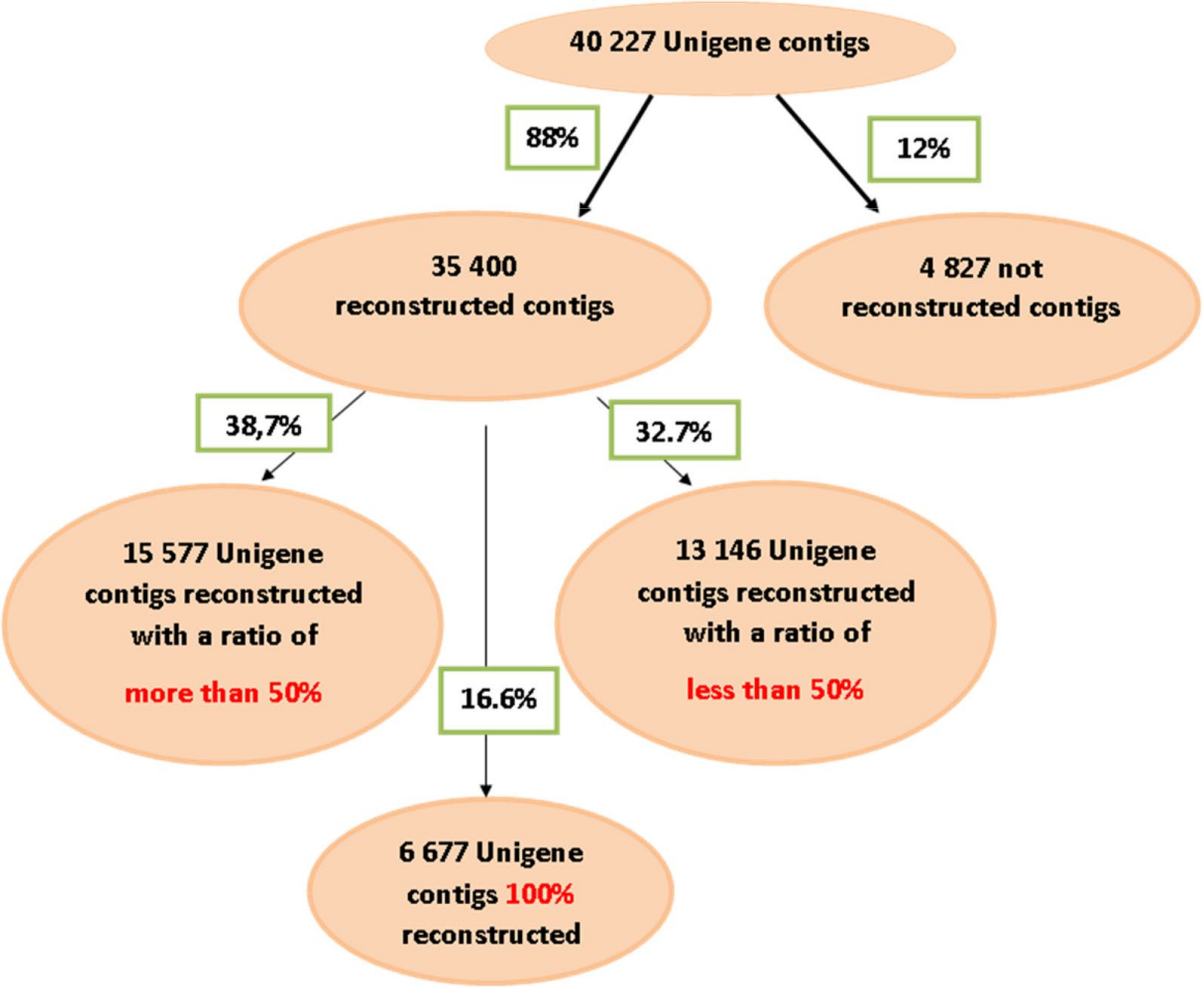
The results of a local BLAST between the 40,227 Unigene contigs allow the estimation of the reconstruction rate of 35,400 de novo contigs at the end of the assembly (iteration I6)

1. The rate of gene reconstruction and the deduction of exon–exon borders on the Unigene sequence was estimated by the results of the standalone BLAST between the Unigene contigs and the de novo contigs reconstructed at the end of the assembly (iteration I6). From the 40,227 contigs that contains the Unigene, 35,400 were found in the de novo assembly (88 %, Fig. 5). This rate indicates that both steps of mapping and assembly did not cause a major loss of information from the initial Unigene. The BLASTN results allow to estimate the reconstruction rate over the 35,400 de novo contigs: 6677 (19 %) were completely rebuilt, 15,577 (44 %) were half to completely rebuilt, and the remaining 13,146 (37 %) were at less than half rebuilt (Fig. 5).

On the 4827 present in the Unigene but not found in the de novo assembly (12 %), nearly half (2100 contigs) do not match against the NCBI database “Nucleotide collection nr/nt” but the other half (2727contigs) matched partially on pea sequence and/or on other species (*Lotus japonicus*, *Cicer arietinum*, *Trifolium repens*, *Vitis vinifera*, *Pinus taeda*) with various degrees of distance to the pea. These contigs could come from the building of chimeric sequences during the assembly.

2. Based on our de novo assembly, the GenoPea 13.2 K SNP genotyping Illumina BeadChip [17] was designed and built to perform gene mapping and genetic variability studies. The repartition of 11,166 SNP with unambiguous position in four groups based on SNP and primer localization in an intron or an exon is presented in Table 1. 762 SNP (6.8 %) are located in an intron and 427 (3.8 %) in an exon, i.e. with probe straddling an exon/intron junction. The genotyping results show that the percentage of polymorphic SNPs detected is similar in the four SNP localizations and around 90 %. The remaining 10 % is divided between non-polymorphic SNPs and SNPs with technical problem. These results validate the good quality of our Gene-space assembly.

**Table 1:** Validation of the four categories of SNP on the GenoPea 13.2 KSNP Illumina genotyping BeadChip

	SNP in exon with primer in exon	SNP in exon with primer partially in intron	SNP in intron with primer in intron	SNP in intron with primer partially in exon
Total number of SNP	7265	762	2712	427
Detected polymorphism	6514 (89.5 %)	685 (90 %)	2442 (90 %)	380 (89 %)
Non-detected polymorphism	551 (7.8 %)	45 (5.9 %)	185 (6.8 %)	33 (7.7 %)
Technical error	200 (2.75 %)	32 (4.2 %)	85 (3.1 %)	14 (3.3 %)

## Discussion

The process described above has allowed us to develop a high-performance genotyping array. These results demonstrate the ability to develop highly effective tools for genomic for species with large genome, mainly because of repetitive sequences, or species where the financial and human resources are limited. This was done by constructing a Gene-space using RNA-seq and DNA-seq data, easily accessible bioinformatic tools and fairly limited computing resources.

The two steps of the protocol were evaluated for their computing time requirements on the hardware specified in the “Methodology” section. The Step-1 of mapping and filtering required about 23 h. The mapping is dependent on the amount of data, their complexity (repeated sequences) and the size of the reference sequence. Our dataset included 1.16 billion reads (122Go) for iteration 1, and 1.06 billion of reads (99.8Go) for the following iterations. The computing time for the mapping in the first iteration was 21 h. The time for data extraction is also directly dependent on the size of the data set. CLC_Assembly_Cell required approximately 2 h to generate the mapping coordinates associated to each read (“.cas” file). The benefits of the filtration Step-1 are visible in the reduction of the computing time and memory requirements in the assembly Step-2. By introducing less complex data, the number of possible branches to be explored during the assembly process is reduced. Step-2 of the assembly performed with HKU-IDBA takes a little more than an hour. The major advantage of the HKU-IDBA is to allow an iterative increase of the kmer parameter between two values, kmin and kmax, thus avoiding an arbitrary selection of the size of kmer used in the assembly. We used a kmin = 20 because a kmer value under 20 presents a greater risk not to be unique, and a kmax = 100 corresponding to the maximum length of reads. We analyzed the influence of different values of the kmin to kmax step increment value (from 1 to 20 nt) on the number and average length of generated contigs and the compatibility with the computer resources available. A step of 5 nt was chosen as the best compromise between quality of assembly and computing time.

The number of iterations to reach the plateau was linked in our study to two parameters: the intron length and the read length. The longer the intron, the higher is the required number of iterations to completely reconstruct the sequence between the two exons. Conversely, longer read decrease the number of iterations needed to reconstruct the sequence. It is usually considered that two third introns are smaller than 150 nt [18]. This can explain why the total number of bases included in the assembly increases by 33 % at the first iteration (Fig. 4a, b). Going past the 6th iteration did not make sense for most of pea genes. However for genes with longer introns, it may be helpful to perform additional iterations by removing the already completed genes from the dataset, in order to decrease computing time.

Our iPEA approach is near to Dutilh et al. [19] for the construction of a bacterial metapopulation consensus genome. The mapping to a reference sequence followed by the assembly of the extracted reads is certainly an attractive approach to allow gene analysis in the case of genotypes or metagenome where the only available reference sequence can be phylogenetically distant. Of course, the quality of this de novo assembly is highly dependent of the mapping of the first iteration, which can be extended onto a Unigene dataset or any previously assembled reference sequence. The criteria of mapping (length and degree of similarity) must be adjusted as not to lose the richness present in the raw data files by a too high stringency. The final assembly performed at the end of the different iterations is a reconstruction of the actual sequences existing in a particular sample.

Finally, the Bioinformatics tools (CLC_ref_assemble_long and sub_assembly, and HKU-IDBA) to perform the mapping and assembly were effective in this work but it is certain that other programs for mapping and assembly can be change into the IPEA pipeline (Fig. 3) to the user convenience.

## Conclusion

The iPEA de novo assembly strategy allows laboratories with limited computer resources to quickly assemble a reference sequence mainly consisting of “Gene-space” using RNA-Seq and DNA-Seq data (now easy to obtain regardless of the species).

While this protocol can’t be considered exhaustive, its main interest is to provide a sequence assembly focusing on the most informative and stable regions of the genome. The results obtained after Illumina genotyping confirmed that the de novo assembly was valid, and that this approach is effective and of interest when reference sequences are absent or of limited value for the plant genotypes under investigation.

The analysis of the variability among genotypes of diverse origins will not be satisfied with comparison with a limited number of reference sequences. It is therefore essential to develop tools and strategy for the assembly of “de novo” sequence based on the actual NGS data and more closely “relevant” to the genetic variability under investigation.

## Additional files

10.1186/s13104-016-1903-z Number of raw and trimmed reads from the different protocols of sequencing.
10.1186/s13104-016-1903-z Mapping of genomics HiSeq 2000 reads against the pea Unigene at the first iteration.
10.1186/s13104-016-1903-z Evolution of the number and the size of the de novo genomics contigs as a function of the iterations.

## Abbreviations

NGS: next generation sequencing
SNP: single nucleotide polymorphism
BLAST: basic local alignment search tool
BLASTN: nucleotide BLAST
