# Supplementary material for: De novo construction of a “Gene-space” for diploid plant genome rich in repetitive sequences by an iterative Process of Extraction and Assembly of NGS reads (iPEA protocol) with limited computing resources
Source: BMC Res Notes. 2016 Feb 11;9:81. doi: 10.1186/s13104-016-1903-z (PMC4750290; doi:10.1186/s13104-016-1903-z)
Supplement: Supplementary file 1 — 10.1186/s13104-016-1903-z Number of raw and trimmed reads from the different protocols of sequencing. [file 13104_2016_1903_MOESM1_ESM.doc]

**Additional file 1:** Number of raw and trimmed pairs from the different protocols of sequencing.

| Sequencer | HiSeq2000 | HiSeq2000 | HiSeq2000 | Total HiSeq2000 | HiSeq2000 | HiSeq2000 | Total HiSeq2000 | Total HiSeq2000 | Total MiSeq |
| --- | --- | --- | --- | --- | --- | --- | --- | --- | --- |
| Flowcell Names | D08JCACXX | COMNEACXX | D0CEUACXX |  | D150JACXX | D14E7ACXX |  |  | M01075_ A1NGY |
| Insert length | 390 | 390 | 390 | 390 | 620 | 620 | 620 |  | 620 |
| Read length | 101 | 101 | 101 | 101 | 101 | 101 | 101 | 101 | 250 |
| Number of pairs | 182 022 364 | 100 060 283 | 68 039 805 | 350 122 452 | 162 626 431 | 49 744 513 | 212 370 944 | 562 493 396 | 28 527 820 |
| Number of trimmed pairs | 169 428 937 | 98 569 023 | 59 412 104 | 327 410 064 | 155 545 566 | 47 913 347 | 203 458 913 | 530 868 977 | - |
| % | 93% | 99% | 87% | 94% | 96% | 96% | 96% | 94% | - |
