# Supplementary material for: De novo construction of a “Gene-space” for diploid plant genome rich in repetitive sequences by an iterative Process of Extraction and Assembly of NGS reads (iPEA protocol) with limited computing resources
Source: BMC Res Notes. 2016 Feb 11;9:81. doi: 10.1186/s13104-016-1903-z (PMC4750290; doi:10.1186/s13104-016-1903-z)
Supplement: Supplementary file 2 — 10.1186/s13104-016-1903-z Mapping of genomics HiSeq 2000 reads against the pea Unigene at the first iteration. [file 13104_2016_1903_MOESM2_ESM.doc]

**Additional file 2:** Statistics of mapping of HiSeq2000 paired end reads against the pea Unigene at the first iteration. Information from three DNA libraries (390 nt insert size) and two DNA librairies (620 nt insert size) runned on five HiSeq2000 flowcells.

| **Insert size of the libraries (nt)** | **390** | **620** |
| --- | --- | --- |
| **Reads lenght (in bp)** | 101 | 101 |
| **Number of pairs** | 327 410 064 | 203 458 913 |
| **Number of reads** | 654 820 128 | 406 917 826 |
| **Number of mapped reads**  **% of mapped reads** | 6 886 914 | 4 168 932 |
|  | **1.0%** | **1.0%** |
| **Multi-hit matched reads**  **% of multi-hit matched reads compared to mapped reads** | 653 664 | 413 991 |
|  | **9.5%** | **9.9%** |
| **Number of reads mapped in paired-end**  **% of reads mapped in paired-end compared to mapped reads.** | 1 804 793 | 533 325 |
|  | **26.2%** | **12.8%** |
| **Sites covered 0 times** | 11 212 641 | 11 653 366 |
|  | **21.8%** | **22.7%** |
| **Average coverage** | **13 X** | **8 X** |
