# Supplementary material for: De novo construction of a “Gene-space” for diploid plant genome rich in repetitive sequences by an iterative Process of Extraction and Assembly of NGS reads (iPEA protocol) with limited computing resources
Source: BMC Res Notes. 2016 Feb 11;9:81. doi: 10.1186/s13104-016-1903-z (PMC4750290; doi:10.1186/s13104-016-1903-z)
Supplement: Supplementary file 3 — 10.1186/s13104-016-1903-z Evolution of the number and the size of the de novo genomics contigs as a function of the iterations. [file 13104_2016_1903_MOESM3_ESM.doc]

**Additional file 3: Evolution of the number and the size of the *de novo* genomics contigs as a function of the iterations.**

|  |  | **Iteration 1** | **Iteration 2** | **Iteration 3** | **Iteration 4** | **Iteration 5** | **Iteration 6** |
| --- | --- | --- | --- | --- | --- | --- | --- |
| **Filtration Step** | Reference sequence name and contigs number for the filtration step | Unigene 40 227 | REF1  56 219 | REF2 47 511 | REF3 42 272 | REF4 41 835 | REF5 40 512 |
|  | Number of reads before the filtration step | 530 868 977 | 530 868 977 | 530 868 977 | 530 868 977 | 530 868 977 | 530 868 977 |
|  | Number of reads after the filtration step | 16 335 096 | 89 531 134 | 36 007 966 | 42 423 498 | 36 569 950 | 43 391 314 |
| **Input Data for the assemby step** | Input "short reads" data from filtration step for HKU-IDBA algorithm | 16 335 096 | 89 531 134 | 36 007 966 | 42 423 498 | 36 569 950 | 43 391 314 |
|  | Input" long reads" sequence name for HKU-IDBA algorithm and contigs number | Miseq data 28 527 820 | REF1 56 219 | REF2 47 511 | REF3 42 272 | REF4 41 835 | REF5 40 512 |
| **Ouput data after the Assembly step** | Output sequence name and number of build contigs | REF1 56 219 | REF2 47 511 | REF3 42 272 | REF4 41 835 | REF5 40 512 | REF6 40 901 |
|  | Residue counts | 81 146 443 | 72 896 231 | 85 518 796 | 90 619 971 | 95 546 587 | 97 275 026 |
|  | Min length contigs (bps) | 299 | 299 | 299 | 299 | 299 | 299 |
|  | Max length contigs (bps) | 18 294 | 10 475 | 18 784 | 18 819 | 19 273 | 18 907 |
|  | Average length (bps) | 1 443 | 1 534 | 2 023 | 2 166 | 2 356 | 2 378 |
|  | N50 (bps) | 1 931 | 2 152 | 2 778 | 3 033 | 3 326 | 3 416 |
